# Histopathologic Validation of the Sentinel Node Technique for Early-Stage Cervical Cancer Patients

**DOI:** 10.1245/s10434-020-09328-2

**Published:** 2020-11-12

**Authors:** Patrice Mathevet, Benedetta Guani, Andrea Ciobanu, Eliane Mery Lamarche, Florent Boutitie, Vincent Balaya, Fabrice Lecuru

**Affiliations:** 1 Department of Gynecology, University Hospital of Lausanne, Lausanne, Switzerland; 2 Department of Medicine, University of Lausanne, Lausanne, Switzerland; 3 Department of Gynecology, Leman Hospital, Thonon Les Bains, France; 4 Department of Pathology, IUCT Oncopole, Toulouse, France; 5 Department of Biostatistics, University Hospital of Lyon, Lyon, France; 6 Paris Descartes University, Paris, France; 7 Department of Gynecology, Curie Institute, Paris, France

## Abstract

**Background:**

The sentinel lymph node (SLN) biopsy may be an alternative to systematic lymphadenectomy in early cervical cancer. The SLN biopsy is less morbid and has been shown to have high sensitivity for metastasis detection. However, the sensitivity of the SLN technique might be overevaluated because SLNs are examined with ultra-staging, and non-sentinel nodes usually are examined only with routine techniques. This study aimed to validate the negative predictive value (NPV) of the SLN technique by the ultra-staging of SLNs and non-sentinel nodes (NSLNs).

**Methods:**

The SENTICOL 1 study data published in 2011 were used. All nodes (i.e., SLNs and NSLNs) were secondarily subjected to ultra-staging. The ultra-staging consisted of sectioning every 200 µm, in addition to immunohistochemistry. Moreover, the positive slides and 10% of the negative slides were reviewed.

**Results:**

The study enrolled 139 patients, and SLNs were detected in 136 (97.8%) of these patiets. Bilateral SLNs were detected in 104 (76.5%) of the 136 patients. A total of 2056 NSLNs were identified (median, 13 NSLNs per patient; range 1–54). Of the 136 patients with SLNs, 23 were shown to have positive SLNs after serial sectioning and immunohistochemical staining. The NSLNs were metastatic in six patients. In the case of bilateral SLN detection, the NPV was 100%, with no false-negatives (FNs).

**Conclusions:**

The pelvic SLN technique is safe and trustworthy for determining the nodal status of patients with early-stage cervical cancer. In the case of optimal mapping with bilateral detection, the NPV was found to be 100%.

In early-stage cervical cancer, lymph node status is one of the most important prognostic factors. The revised International Federation of Gynecology and Obstetrics (FIGO) 2018 classification,^1^ which specifically defines lymph node involvement as stage 3C disease, highlights the importance of lymph node metastasis as a major prognostic factor in cervical cancer. In contrast to macrometastases (MACs) and micrometastases (MICs), the presence of isolated tumor cells (ITCs) does not imply a stage change.^1^ Whereas MACs are lymph node metastases larger than 2 mm, MICs are lymph node metastases larger than 0.2 mm, and ITCs are tumor cell clusters smaller than 0.2 mm.

For a long time, lymph node status was assessed by pelvic lymphadenectomy because pelvic lymph node dissection increased postoperative morbidity.^2^ For this reason, the sentinel lymph node (SLN) technique has been introduced more recently, and several studies have demonstrated its feasibility and reliability.^3^^–^5 In the 2015 National Comprehensive Cancer Network (NCCN) guidelines,^6^ SLN mapping is considered to be an alternative to pelvic lymphadenectomy (category 2B).

The SLN technique is a sensitive method for detecting lymph node involvement. Its sensitivity and negative predictive value (NPV) are high (99%) if the SLNs are detected bilaterally.^3^^,^^7^^,^^8^ Ultra-staging improves the sensitivity of SLN biopsies because MICs and ITCs are detected in a substantial number of cases. The combination of surgical mapping and ultra-staging results in a higher rate of positive lymph nodes detected, especially compared with routine lymphadenectomy and routine pathology.^9^ However, the clinical significance of MICs and ITCs currently is controversial. Despite encouraging results in recent prospective studies,^10^^–^12 criticism has been levied against the diagnostic accuracy of the SLN technique, with the assertion that only SLNs undergo ultra-staging, whereas non-SLNs (NSLNs) are examined with a single slide in a routine fashion. The ultra-staging of SLNs (and not NSLNs) could artificially enhance the sensitivity of the technique.

To assess the NPV of the sentinel node technique, we analyzed the results of the SENTICOL 1 study^3^ after ultra-staging was performed for all nodes using SLN and NSLN. The SENTICOL 1 study is a prospective multicenter investigation of early cervical cancer patients treated with radical surgery and SLN biopsy plus pelvic lymph node dissection that identifies SLNs with a combined (colorimetric and isotopic) detection method.^3^

The goal of this study was to assess the NPV of the SLN technique, including the identification of low-volume metastases in the SLN. This report presents the results of ultra-staging in all sentinel and NSLNs. To perform this precise evaluation, every retrieved lymph node, SLN, and NSLN were extensively studied with serial sectioning every 200 µm, and immunohistochemistry was applied with an anti-cytokeratin antibody to identify MICs and ITCs.

## Materials and Methods

### Patient Selection

A prospective, multicentric longitudinal study (SENTICOL 1) was conducted in seven French centers between 1 January 2005 and 30 June 2007. The study was approved by the appropriate ethics committee (“Comité de Protection des Personnes,” HEGP, Broussais), supported by clinical research grant no. AOR 03063 to the National Hospital Clinical Research Project, 2003 by the French National Cancer Institute (INCA), Boulogne Billancourt, France, and by the Programme Hospitalier de Recherche Clinique (PHRC) and Soutien aux Technologies et Innovations coûteuses (STIC).

Consecutive patients were enrolled prospectively for evaluation of the sentinel node. Consecutive patients were prospectively enrolled for evaluation of the SLN technique with a double-labeling method as follows:

$$ \begin{aligned} {\text{Isotopic}}\;{\text{method}} & = {\text{Nanocis}}\;{\text{injection}} \\ {\text{Colorimetric}}\;{\text{method}} & = {\text{Patent}}\;{\text{Blue}}\;{\text{injection}}. \\ \end{aligned} $$

We included adults with cervical carcinoma who met the International Federation of Gynecology and Obstetrics (FIGO) 2009 criteria for stage 1A1 disease with lymphovascular space invasion and stage 1B1 disease with squamous, adenocarcinoma, or adenosquamous histology. Written informed consent was obtained from all the patients before their inclusion in the study.

### Surgical Methods

All the patients prospectively underwent a laparoscopic SLN biopsy with full pelvic lymph node dissection and radical hysterectomy or radical trachelectomy. Each node that was blue or “hot” as well as blue and “hot” was considered as an SLN. The detailed surgical procedure has already been described in a previous article.^3^

### Histopathologic Examination of the SLNs and NSLNs

We assessed the NPV of the SLN biopsy by studying SLNs and NSLNs with the same technique. All the nodes (SLNs and NSLNs) were evaluated with ultra-staging.

All sentinel lymph nodes were bisected if larger than 4 mm and processed in individual paraffin blocks. Then, each block underwent serial sectioning every 200 µm, and all the slides were stained with hematoxylin–eosin–saffron (HES). When the staining was negative, a section from the same level was examined using immunohistochemistry (IHC) with the pan-cytokeratin antibody AE1–AE3 (1:500 dilution; DAKO, Trappes, France) to identify tumor deposits. If the node was found to be metastatic after HES staining, IHC with the pan-cytokeratin antibody analysis was not performed. For SLNs, all the sections were analyzed at the centers where they were taken by the same experienced pathologists at said centers.

The NSLNs were processed by routine surgical pathology techniques at each center. The nodes were bisected if larger than 4 mm and processed in a paraffin block. One level was stained with HES in each center. Subsequently, every NSLN block was sent for ultra-staging at one center for serial sectioning and processed in the same manner as the SLNs. Each block underwent serial sectioning every 200 µm, was stained with HES, and eventually was analyzed by IHC with the pan-cytokeratin antibody AE1–AE3 (1:500 dilution; DAKO). All the sections from all the patients were analyzed by the same experienced pathologist.

A central reviewing of the positive or doubtful slides and 10% of the negative slides was performed by four expert pathologists [E. Mery Lamarche (co-author), A. Burnerd, M. C. Baranzelli, and P. Duvillard (in the acknowledgments)].

### Statistical Methods

The primary objective of the SENTICOL 1 study^3^ was to determine the sensitivity and NPV of the SLN biopsy. We performed an ultra-staging evaluation of all nodes from the pelvis, with or without para-aortic lymphadenectomy.

We performed a modified intention-to-diagnose (mITD) analysis of the patients who underwent SLN detection and determined the rate for SLN detection. When computing diagnostic performance parameters, we excluded patients in whom no SLNs were detected. Given that false-positive results could not exist, we did not compute the specificity, positive predictive value, or likelihood ratios. All the 95% confidence intervals (CIs) for the proportions were estimated using the exact binomial distribution.

Univariate analyses were conducted using the Chi square test or Fisher’s exact test for categorical variables and logistic regression models for continuous quantitative variables. The final multivariate logistic regression model was obtained using a stepwise selection of the factors significant at the 0.05 level in the univariate analysis.

All *p* values lower than 0.05 were considered significant. All analyses were performed using SAS (release 9.1; SAS Institute, Cary, NC, USA).

## Results

This study enrolled 144 patients in seven centers. Two patients with a history of cancer (endometrial cancer and pelvic lymphadenectomy for colon cancer, respectively) were excluded, and four patients left the study before the SLN detection procedure. Consequently, 139 patients remained in the final mITD population. This population included 11 patients with major protocol deviations, 6 of whom did not receive the isotope injection. One patient received an injection of an isotope different from Nanocis. The clinicopathologic characteristics of the 139 patients are presented in Table 1.

**Table 1:** Main clinical patient and disease characteristics

Clinical characteristics	No. of patients (%)
Median age: years (range)	43 (23–85)
Mean BMI: kg/m^2^ (range)	22 (15–45)
Histology	
Squamous carcinoma	103 (74.1)
Adenocarcinoma	34 (24.4)
Adenosquamous carcinoma	2 (1.4)
FIGO stage 2008	
1A1 + LVSI	5 (3.6)
1A2	12 (8.6)
1B1	121 (87.1)
2A	1 (0.7)
Median largest tumor diameter: mm (range)	13 (0–43)

*BMI* body mass index, *FIGO* International Federation of Gynecology and Obstetrics, *LVSI* lymph-vascular space involvement

All 139 patients received a Patent Blue injection, and 132 patients received the isotope tracer, Nanocis. All the patients underwent biopsies for all identified SLNs, followed by pelvic, para-aortic lymphadenectomy, or both. The analysis was performed per patient and also per pelvic side of detection. A total of 139 patients and 278 (139 × 2) hemi-pelvises were analyzed.

In all, 454 SLNs were identified, with no significant difference between sides (*p* = 0.44). The median number of SLNs was three per patient (range 0–10) and one per side (range 0–6). In the pelvic lymphadenectomy specimens, with or without para-aortic lymphadenectomy, 2056 NSLNs were identified (median number per patient, 13; range 1–54).

Sentinel lymph nodes were detected in 136 (97.8%) of the 139 patients, and 104 (76.5%) of the 136 patients had SLNs on both sides. Of the 136 patients with detected SLNs, 23 had positive SLNs.

After serial sectioning, IHC, and central reviewing, SLNs were found to be metastatic in 23 patients, whereas NSLNs were metastatic in 6 patients (Table 2).

**Table 2:** Early cervical cancer patients with a metastatic NSLN after serial sectioning (SS) and immunohistochemistry

Patient	Side	SLN	NSLN	Comment
1	R	MIC	MAC	
	L	MIC	Neg	
2	R	MAC	Neg	
	L	MAC	MAC	
3	R	–	ITC	Bilateral failure of SLN detection
	L	–	MAC	
4	R	–	MIC	Failure of right SLN detection and MIC on the right side
	L	Neg	Neg	
5	R	Neg	Neg	
	L	ITC	MIC	
6	R	Neg	ITC	Failure of left SLN detection False-negative case on the right side
	L	–	Neg	

*NSLN* non-sentinel lymph node, *SLN* sentinel lymph node, *R* right, *L* left, *MIC* micrometastasis, *MAC* macrometastasis, *Neg* negative, *ITC* isolated tumor cells

The central reviewing showed only one discordant case. This was a case of adenocarcinoma with a negative HPV and a doubtful result of the SLN. The lymph node showed inclusions (benign vs. metastatic), and by consensus of the pathologic experts, the SLN was classified as positive.

Two of the six patients with metastatic NSLNs had positive SLNs on both sides of the pelvis and also positive NSLNs. Therefore, the ultra-staging of the NSLNs added no information.

Although one patient had no SLNs detected, one ITC and one MAC were found in the pelvic NSLN. This patient was considered as having SLN detection failure.

One patient had unilateral SLN detection, and NSLN ultra-staging showed one MIC in one pelvic node located on the pelvic side without SLN detection. The other side of the pelvis had no nodal extension (SLN or NSLN). This patient was considered a detection failure case per pelvic side.

One patient had one positive SLN on the left side of the pelvis (ITC) and a negative SLN on the right side of the pelvis, and NSLN ultra-staging showed one left pelvic lymph node with MIC. This patient was considered a true positive case per pelvic side.

The last patient had an SLN detected on one side of the pelvis. This SLN was negative. The final analysis showed one ITC in one NSLN on the same side as the negative SLN. This patient was considered a failure case in terms of left SLN detection and a false-negative case per pelvic side.

For the 32 patients with unilateral SLN detection, the false-negative rate was 3% (1/32), with the detection of one ITC in an NSLN despite a negative SLN in the same hemi-pelvis. The NPV was 97% [95% CI 0.80–1.00)].

Of the 104 patients with bilateral SLN detection, 3 (patients 1, 2, 5 in Table 2) who had both positive SLN and positive NSLN. For the patients with bilateral SLN detection, the false-negative rate was 0 (0/104), with an NPV of 100%.

## Discussion

The last important benefit of the SLN technique is the possibility of omitting the full regional lymph node dissection in the case of a negative SLN. This usually is recommended to reduce the risk of pre- and postoperative complications and sequelae.^13^

This prospective study was set up to study the false-negative rates for the SLN technique in early cervical cancer. In the SENTICOL 1 study,^3^ patients were prospectively evaluated for SLNs with a combined (isotopic and Patent Blue) technique. Afterward, they underwent a full pelvic lymph node dissection.

To obtain a more detailed and precise picture of the SLN technique’s accuracy, we performed an extensive SLN evaluation. In a second step to identify small tumor deposits, we evaluated NSLNs with serial sectioning and IHC. All sections from all the patients were analyzed by the same experienced pathologist. A central review of the positive and doubtful slides and 10% of the negative slides was performed by four expert pathologists. The goal of this study was to validate the SLN biopsy technique from the low-volume metastasis point of view.

The ability to detect metastatic tumoral cells in the lymph nodes is directly correlated with the extent of lymph node dissection and histopathologic techniques used for lymph nodal evaluation. Detecting low-volume metastases (MICs and ITCs) in lymph nodes requires serial sectioning (the usual thickness between two sections is 200 µm, the minimal size of the micrometastasis) and IHC. Use of IHC with a cytokeratin antibody helps in detecting small tumoral implants that would not be identified with routine hematoxylin and eosin (H&E) staining. However, cytokeratin IHC may detect benign epithelial inclusions that are quite frequent in pelvic nodes and should be differentiated from tumoral implants.^14^

Five patients (3.5%) in our study had benign epithelial inclusions diagnosed by serial sectioning and IHC. These ultra-staging techniques combining serial sectioning and IHC improved the rate of nodal metastasis detection and showed 8.1% of apparently node-negative patients to be node-positive.

For the SLN technique to be considered safe, we had to be sure that the NPV of the SLN technique was 100%. Analysis of the relevant literature showed that the false-negative rate for the SLN technique in cervical cancer is low when classical histologic evaluation and H&E staining are performed. In numerous studies, SLNs have been assessed with ultra-staging, whereas screening for NSLNs have been only by routine examination. This favored the apparent diagnostic accuracy of the SLN technique, especially in terms of sensitivity. To evaluate the NPV of the SLN technique fully in early cervical cancer, we set up this prospective study with a double SLN detection technique, complete with SLN and NSLN ultra-staging.

Few studies have evaluated the nodal status of all pelvic nodes with serial sectioning and IHC. Table 3 presents a review of these studies.^15^^–^19 All the studies used the anti-cytokeratin antibody to detect metastatic tumoral cells. However, differences between the studies are observed concerning the level of serial sectioning (i.e., the thickness of the sections varying between 40 and 250 µm) and the techniques for SLN identification.

**Table 3:** SLN studies in early cervical carcinoma after evaluation of the nodal status of all nodes with immunohistochemistry (IHC) and serial sectioning (SS)

Study (year)	No. pat.	FIGO stage	Rate of nodal metastasis (HES) (%)	Rate of nodal metastasis (IHC + SS) (%)	Thickness of the SS (µm)	NPV
Barranger et al.^15^	18	1A2–2B	11	28	150	100%
Marchiolé et al.^16^	29	1A1 + LVSI–1B1	10	28	200	87.5%
Popa et al.^17^	36	1A–2A	0	0	40	100%
Okamoto et al.^18^	10	1B1–2A	0	20	200	100%
Cibula et al.^19^	17	1B–2A	ND	15	150	100% for MIC and MAC
Current study (2020)	139	1A1 + LVSI–1B1	8	19	200	99% in bilateral detection

*No. pat* no. of patients, *FIGO* International Federation of Gynecology and Obstetrics, *HES* hematoxylin–eosin–saffron, *NPV* negative predictive value, *LVSI* lymph-vascular space involvement, *MIC* micrometastasis, *MAC* macrometastasis

In a 2016 study by Cibula et al.,^19^ after processing of all pelvic SLNs by pathologic ultra-staging, no false-negative cases of positive NSLNs (MACs or MICs) or negative SLNs were found. Sentinel lymph node ultra-staging exhibited 100% sensitivity for the presence of both MACs and MICs in pelvic SLNs. The study had one side-specific false-negative case for the presence of ITC in a patient with a negative ipsilateral SLN, but MIC in an SLN and NSLN was found on the other side of the pelvis.

The current study is the most important prospective series to evaluate the SLN technique from a low-volume metastasis point of view. Because patients with low-stage cervical cancer have a low risk of lymphatic invasion, we had only 23 positive SLNs and 6 positive NSLNs. To have more positive NSLNs, it would be necessary to have a much larger sample of patients. Even if the series presented in Table 3 were not similar in terms of the methods used and the numbers of patients, it still appears that the SLN technique is reliable in early cervical cancer, with a high NPV. These results may lead to an evolution of the surgical management for early cervical cancer, with SLN biopsies performed and full pelvic lymph node dissection omitted in the case of negative SLNs to decrease the surgical morbidity related to extensive lymph nodal dissection.

## Conclusion

Our data confirm that after extensive NSLN ultra-staging in early cervical cancer patients, the SLNs identified by precise operative combined techniques and evaluated with serial sectioning and IHC are the lymph nodes most likely to harbor metastatic deposits. Normally MICs, especially ITCs, are missed by classical pathologic analysis. With ultra-staging of all the nodes in this study, we could confirm an NPV of 100% in case of bilateral SLNs detection, also from the point of view of low-volume metastasis (MICs and ITCs).

In conclusion, the pelvic SLN technique is a safe and trustworthy technique for determining the nodal status with an NPV of 100% for patients with early-stage cervical cancer in case of optimal mapping with bilateral detection.

## Appendix

See Table 4.

**Table 4:** Type of sentinel lymph node involvement

Patient	SLN involvement
1	Bilateral MAC
2	2 MIC, ITC
3	1 MIC
4	ITC
5	1 MIC
6	Bilateral MAC
7	1 MAC
8	1 MAC
9	1 MAC
10	ITC
11	1 MAC
12	ITC
13	1 MAC
14	ITC
15	1 MAC
16	ITC
17	1 MIC, ITC
18	ITC
19	1 MIC
20	1 MIC
21	2 MIC
22	1 MAC
23	1 MAC

23 Patients had a positive sentinel lymph node

*SLN* sentinel lymph node, *MAC* macrometastasis, *MIC* micrometastasis, *ITC* isolated tumor cells
